# Roles of the NR2F Family in the Development, Disease, and Cancer of the Lung

**DOI:** 10.3390/jdb12030024

**Published:** 2024-09-10

**Authors:** Jiaxin Yang, Wenjing Sun, Guizhong Cui

**Affiliations:** 1 Department of Basic Research, Guangzhou National Laboratory, Guangzhou 510005, China; yang_jiaxin@gzlab.ac.cn; 2 School of Basic Medical Sciences, Guangzhou Medical University, Guangzhou 511436, China; 2023210098@stu.gzhmu.edu.cn

**Keywords:** NR2F family, lung, carcinogenesis, nuclear receptors

## Abstract

The NR2F family, including NR2F1, NR2F2, and NR2F6, belongs to the nuclear receptor superfamily. NR2F family members function as transcription factors and play essential roles in the development of multiple organs or tissues in mammals, including the central nervous system, veins and arteries, kidneys, uterus, and vasculature. In the central nervous system, NR2F1/2 coordinate with each other to regulate the development of specific brain subregions or cell types. In addition, NR2F family members are associated with various cancers, such as prostate cancer, breast cancer, and esophageal cancer. Nonetheless, the roles of the NR2F family in the development and diseases of the lung have not been systematically summarized. In this review, we mainly focus on the lung, including recent findings regarding the roles of the NR2F family in development, physiological function, and cancer.

## 1. Introduction

Nuclear receptors (NRs), a family of evolutionarily conserved proteins, are ligand-activated transcription factors that participate in the regulation of both physiological and pathological processes [1]. In humans, 48 NRs have been identified, including receptors for steroid hormones, thyroid hormones, cholesterol metabolites, and lipophilic vitamins. NRs are categorized into seven classes: Class 0: miscellaneous; Class I: thyroid hormone receptor-like; Class II: retinoid X receptor-like; Class III: estrogen receptor-like; Class IV: nerve growth factor IB-like; Class V: steroidogenic factor-like; Class VI: germ cell nuclear factor-like [2]. NRs share common structural characteristics, including a transactivation region, a central DNA-binding domain, a region responsible for nuclear localization, and a ligand-binding domain. They function as transcription factors and regulate the expression of genes involved in metabolism, fertility, immunity, angiogenesis and other biological processes [3]. The Nuclear Receptor Subfamily 2 Group F (NR2F) family belongs to Class II of the nuclear receptor superfamily. Due to the lack of identified endogenous ligands, NR2F family members are also known as orphan nuclear receptors.

In humans, the main members of the NR2F family include NR2F1, NR2F2, and NR2F6. NR2F1 and NR2F2 are also named COUP-TFI (Chicken Ovalbumin Upstream Promoter Transcription Factor I) and COUP-TFII (Chicken Ovalbumin Upstream Promoter Transcription Factor II), respectively [4,5,6]. NR2F1 and NR2F2 contain two highly conserved domains, the DNA-binding domain and the ligand-binding domain. NR2F1 and NR2F2 are highly conserved across vertebrate species (in many cases, the conserved subdomains exceed 95% homology) [5]. In general, the NR2F family members exert their functions through two major mechanisms. One is direct regulation by binding to DNA elements, including direct repeat-1, which directly suppresses or activates the expression of target genes. The other mechanism is indirect regulation by interacting with transcription factors such as SP1 to activate the expression of target genes [7,8,9]. NR2F family members perform regulatory functions by forming homodimers or heterodimers. In addition to self-dimerization, NR2F family members also competitively bind with other nuclear receptors, such as retinoic X receptors (RXRs), to inhibit the function of other nuclear receptors [10]. Consequently, several nuclear receptors, such as thyroid hormone receptors (TRs) and retinoic acid receptors (RARs), have been shown to have crosstalk with NR2F family members [10,11].

Previous studies have shown that the NR2F family plays pivotal roles in mammalian embryonic development. For example, in the central nervous system (CNS), *Nr2f1* orchestrates the regionalization of neocortex [12]; meanwhile, both *Nr2f1* and *Nr2f2* are involved in the development of cortical interneurons and the generation of the dorsal–ventral axis of the hippocampus [13,14,15]. Moreover, several studies have demonstrated that mutations in *NR2F1* lead to Bosch–Boonstra–Schaaf optic atrophy syndrome (BBSOAS), which has various symptoms, such as optic atrophy, autism, mental retardation and epilepsy [16,17,18]. It is noteworthy that *Nr2f1* and *Nr2f2* often have a complementary effect on neuronal development. Additionally, *Nr2f2* regulates vasculogenesis in the heart and spinal cord, as well as the development of the kidney, stomach, and diaphragm [19,20,21]. *Nr2f6* is involved in adipocyte differentiation, and it is also considered an essential factor in immune checkpoint regulation to manipulate the development and physiological functions of immune cells [22,23].

Numerous reports have suggested that the NR2F family members are highly involved in cancer, including breast cancer, prostate cancer, and liver cancer [24,25,26,27,28]. Dysregulated long noncoding RNAs associated with the NR2F family have been identified in cancers. For example, *NR2F1* interacted with *NR2F1-AS1* to activate the Sonic Hedgehog signaling pathway and promote the progression of esophageal squamous cell carcinoma [29]. The functions of the NR2F family in CNS development have been reviewed [17,30]. Nevertheless, the roles of the NR2F family in cancer occurrence and progression still lack in-depth studies and systematic summaries. In this review, we summarize the current understanding of the NR2F family in lung development and pathological conditions, proposing an updated and critical view of the various functions of NRs.

## 2. NR2F Family in Lung Development and Non-Cancerous Diseases

**Figure 1:**
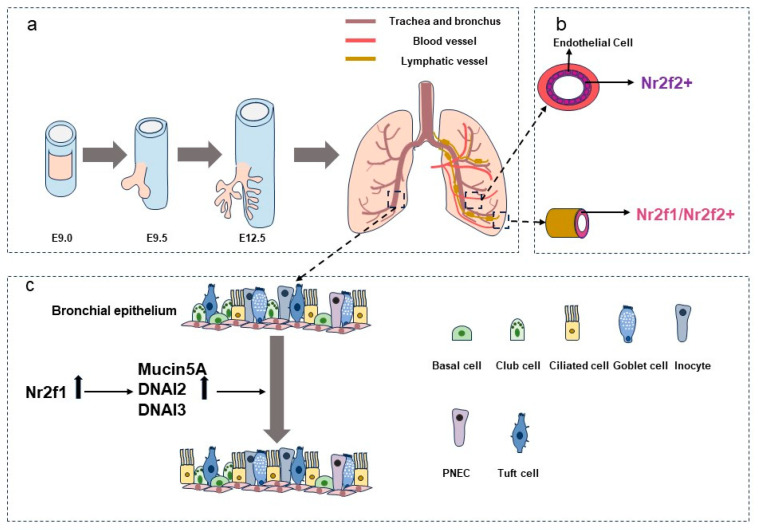
Roles of the NR2F family in the lung development. (**a**) An illustration of lung development. (**b**) NR2F1/2 were identified as markers of angiogenesis and lymphangiogenesis in lung. (**c**) Up-regulation of *Nr2f1* increases the number of lung bronchial epithelial ciliated cells through cilia-related genes such as *DNAI2*.

In mice, lung development begins at E9.0. By E9.5, lung progenitors form the trachea and buds, progressing through stages to generate functional lungs [31] (Figure 1a). Multiple genes regulate lung development. For instance, *Fgf10* regulates early branching morphogenesis [32,33,34]. *Sox2* and *Sox9*/*Id2* dominate the proximal–distal axis patterning. Proximal cells with high *Sox2* expression develop into neuroendocrine cells and non-neuroendocrine cells, while distal cells with high *Sox9/Id2* expression give rise to type I and type II alveolar cells. Alveolar cells are responsible for gas exchange, morphology maintenance, and surfactant secretion [31,35,36]. Abnormalities in terms of lung development can cause diseases like bronchopulmonary dysplasia [37].

Previous studies have demonstrated that NR2F2 is widely expressed in the developing lung [38]. With advancements in single-cell RNA sequencing and the stem cell-derived organoid system, NR2F1 has been shown to be expressed in the foregut and developing lung epithelium and mesenchyme [39]. Both blood vessels and lymph vessels are essential components of the lung mesenchyme. Recent studies suggest that the NR2F family may play critical roles in lung angiogenesis and lymphangiogenesis. *NR2F1* and *NR2F2* have been identified as lymphatic marker genes, with *NR2F2* specifically marking venous endothelial cells [40] (Figure 1b). Additionally, *NR2F1* has been suggested in a BioRx preprint to be one of the genes involved in the core organ-size regulation program, displaying a unique expression pattern in the developing swine lung epithelium and mesenchyme [41]. In the lung epithelium, the expression of *NR2F1* is restricted to the initial stages of lung development, whereas it is almost absent in later stages. In contrast, in the lung mesenchyme, *NR2F1* is continuously expressed throughout development. Furthermore, a function of *Nr2f1* in the growth and differentiation of ciliated bronchial epithelium was uncovered in a study evaluating the role of *Pten* overexpression in lung cancer [42]. *Pten* overexpression blocked this function of *Nr2f1*. These authors also found that *Nr2f1* upregulated other ciliogenesis-related genes, including *Mucin5a*, *DNAI2*, and *DNAI3* (Figure 1c). Despite some progress in understanding the role of the NR2F family in lung development, the regulatory mechanisms remain largely unexplored. Nonetheless, the association of the NR2F family with various lung-related diseases underscores its significant functions in the lung.

Congenital diaphragmatic hernia (CDH) is a severe lung-related developmental disease with an incidence rate of approximately 1/3000 and a mortality rate exceeding 30%. Several studies suggest that *NR2F2* deficiency induces CDH [21,43]. Moreover, pulmonary fibrosis is a progressive lung disease characterized by fibrosis and scar formation in the distal lungs. Idiopathic pulmonary fibrosis (IPF) is the most common form of pulmonary fibrosis without effective treatment available to date. Several studies demonstrated that *Nr2f2* can affect IPF by influencing downstream genes such as *Col1a1* and *Fn1*, inhibiting the activation of fibroblasts and the production of extracellular matrix, and enhancing the dissolution of fibrosis [44,45]. Lymphangioleiomyomatosis (LAM), another lung disease, is characterized by abnormal proliferation of smooth muscle, which leads to the obstruction of pulmonary bronchioles and lymphatics, as well as lung function impairment, including pneumothorax. Recent research indicates the potential roles of *NR2F2* in the progression of LAM due to its overexpression in tumor tissues [46].

## 3. NR2F Family in Primary Lung Cancer

Lung cancer is the leading cause of cancer-related deaths worldwide. Lung cancer can be categorized into small-cell lung cancer and non-small-cell lung cancer (NSCLC). Small-cell lung cancer, characterized by rapid growth and high metastatic potential, is less common but predominantly found in smokers, with most patients exhibiting TP53 mutations [47,48,49]. NSCLC, which accounts for over 85% of all lung cancer cases, can be further classified into lung squamous cell carcinoma (LUSC), lung adenocarcinoma (LUAD), and large cell carcinoma. LUSC and LUAD have been prevalent and extensively studied [47]. LUSC originates mainly from the internal epithelial cells of the bronchi or bronchioles, and it is characterized by a squamous cell morphology, keratinization, and the presence of intercellular bridges [50]. LUAD arises from glandular cells with secretory functions in the lungs and exhibits diverse morphological features, and it can be identified by NKX2.1 expression or Napsin-A staining [47].

Studies on LUAD have found that the overexpression of *NR2F1* can enhance the migration and invasion of tumor cells, probably through *NR2F1-AS1*, which is upregulated by *NR2F1* and *ZEB1* [51]. Intriguingly, the overexpression of *NR2F2* in lung tumor cells also enhances their invasion and migration capabilities by in vitro modeling [52]. Furthermore, *NR2F2* is regulated by the Wnt signaling pathway to activate the expression of *GPX4*, which could induce high glutathione (GSH) consumption to inhibit ferroptosis and lead to the drug resistance of lung cancer cells that metastasize to the brain [53]. Additionally, *NR2F6* expression is significantly upregulated in LUAD tissue [54], and the single nucleotide variation of *NR2F6* is strongly related to the survival rate of patients in the early stage of NSCLC [55]. These results from lung and other tissue cancer studies suggest that *NR2F6* plays important roles in immunity, metabolism, and the reaction of T cell responses to inflammatory cytokines, such as IL2 and TNFβ, which mediate anti-cancer immune reactions [23,56] (Table 1).

## 4. NR2F Family in Metastatic Lung Cancer

Most cancer-related deaths result not from the primary tumor itself but from metastatic dissemination [60]. In the later stages of cancer, primary tumor cells undergo transformation, then travel to distant sites, and re-establish tumor clones. Almost any cancer can spread to the lungs since all blood must pass through the lungs during oxygenation and any circulating tumor cell could be filtered out in its rich capillary network. Many cancer patients in advanced stages are often discovered to have lung lesions, particularly in patients with breast and colon cancer, which are highly prone to lung metastasis. The late-stage metastasis of tumor cells is an important factor contributing to the challenge of the treatment and the high mortality rate. The process of tumor cell metastasis to the lungs involves several stages, including tumor cells detaching from the primary tumor tissue, infiltrating surrounding tissues, invading the blood or lymphatic vessels, entering the lungs through the bloodstream and lymphatics, extravasating from the vessels, colonizing in lung tissue, initiating growth, and eventually forming metastatic lung cancer [61,62].

During tumor cell metastasis, several crucial biological processes unfold, including reshaping of the tumor microenvironment (TME), transformation of the tumor cell status, and the dormancy and activation of tumor cells [61,62,63,64]. Reshaping the TME primarily involves the activation of inflammatory responses, increased angiogenesis, and immune suppression [63]. The transformation of the tumor cell status includes the transition of tumor cells from an epithelial cell state to a mesenchymal cell state, known as epithelial–mesenchymal transition (EMT), during the initial stages of metastasis, facilitating migration and invasion. Subsequently, upon reaching distant organs via the bloodstream, tumor cells may undergo mesenchymal–epithelial transition (MET), reverting to an epithelial state to support rapid proliferation [61,62,63]. Upon initial arrival in the lungs, tumor cells often enter a period of dormancy before being reactivated, which is possibly related to the establishment of a new niche of tumor cells in the lungs, and it is also a significant reason why many cancer patients experience recurrence after undergoing curative treatment [61,64].

Studies using animal models of metastatic lung cancer indicate that elevated *NR2F1* expression in tumor cells can induce dormancy in lung tissues by co-regulating with *SMAD4* and *TGFβ*, causing tumor cells to exit the cell cycle [65]. Similarly, *NR2F1-AS1* upregulates *NR2F1* expression to suppress *ΔNp63* expression and prevent the MET process in tumor cells, leading to reduced proliferation of breast cancer cells that have metastasized to the lungs [66].

In both human tissues and cellular models, *NR2F1* suppresses the metastasis of salivary adenoid cystic carcinoma (SACC) tumor cells to the lungs by upregulating the CXCL12/CXCR4 pathway [67]. *Nr2f2* modulates the metastasis of breast tumor cells to the lungs by activating the expression of *Ang1*, thereby promoting tumor angiogenesis, facilitating the provision of nutrients and oxygen to support tumor cell metastasis to the lungs [24]. Additionally, reports on gastric cancer with lung metastases have discovered that *Fbxo21* inhibits EMT by suppressing *Nr2f2* in both in vivo tissues and in vitro cell lines [68]. These findings underscore the involvement of the NR2F family in the metastatic processes of various tumor cells in relation to the lungs, which could indicate its significance in the progression of metastatic lung cancer (Table 2).

## 5. Other Members of the Nuclear Receptor Superfamily Associated with the NR2F Family and Lung Cancer

**Figure 2:**
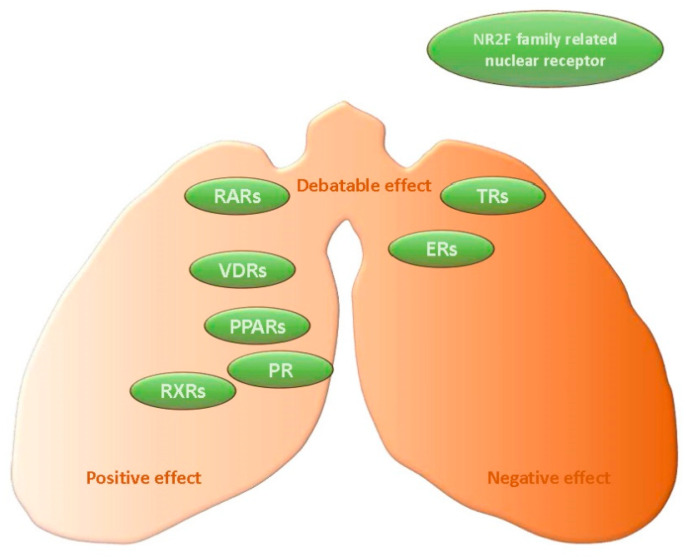
The effect of nuclear receptors in lung cancer. These seven nuclear receptors are all associated with the NR2F family, and the nuclear receptors near the left of the figure tend to have a positive effect in lung cancer, while the nuclear receptors near the right of the figure tend to have a negative effect, and the nuclear receptors near the middle of the figure have a debatable effect.

In addition to the NR2F family, there are more than 40 members of the nuclear receptor superfamily [70], many of which play important roles in organ development and homeostasis, including the lungs. These nuclear receptors actively regulate various cellular functions; in addition, the expression levels of many nuclear receptors, such as progesterone receptor (PR), have been identified as prognostic factors for lung cancer patients [71,72]. The NR2F family members either interact with other nuclear receptors, such as RXRs, to form heterodimers or compete with other nuclear receptors for the binding sites of target genes to mutually regulate their functions [10]. Therefore, summarizing the roles of other nuclear receptors in lung cancer can provide further insights into their interaction mechanisms with the NR2F family (Figure 2).

### 5.1. Estrogen Receptors (ERs)

ERs belong to Cass III of the nuclear receptor superfamily and serve as receptors for the steroid hormone estrogen. ERs, including two subtypes ERα and ERβ, play essential roles in normal cell growth, differentiation, and survival [70]. Several reports have revealed a close association between ERs and *NR2F2* expression. *NR2F2* is highly expressed in ER-positive breast cancer cell lines but is poorly expressed in ER-negative breast cancer cell lines [73]. Additionally, *Nr2f1* can also modulate the activity of ERs [74]. Studies in non-small-cell lung cancer have shown the dynamic expression of ERs, indicating that ERs could potentially have diverse functions in the genesis and progression of lung cancer [75,76,77,78]. Treatment with ER agonists have been found to increase the proliferation of lung tumor cells in animal models, while ER antagonists inhibit cell growth through IL-6 [79].

### 5.2. Progesterone Receptor (PR)

Similar to ERs, PR belongs to Class III of the nuclear receptor superfamily and is a receptor for progesterone. PR has two isoforms, PR-A and PR-B, which form homodimers or heterodimers to bind to the progesterone response elements (PREs) on DNA and to regulate the expression of target genes [80]. In breast cancer cell lines, PR and ERs collaborate to downregulate the transcription of *NR2F1-AS1* [81]. During embryonic implantation, PR regulates the expression of *NR2F2* by controlling *Indian Hedgehog*, which can activate *NR2F2,* then *NR2F2* inhibits ERs in the uterine epithelium [82]. Several studies have shown a significant decrease of PR in lung cancer tissues [83], and similar results were observed in a mouse model with lung tumor cells transplanted [84], suggesting that PR could be a potential target for lung cancer treatment.

### 5.3. Retinoic Acid Receptors (RARs)

RARs belong to Class I of the nuclear receptor superfamily and act as receptors for retinoic acid. RARs, which can be classified into three subtypes, RARα (*NR1B1*), RARβ (*NR1B2*), and RARγ (*NR1B3*), regulate cell proliferation, differentiation, and death [70]. Previous studies indicated that the NR2F family members inhibit the target gene regulation of RARs [10]. Intriguingly, *NR2F1/2* can be activated by RA signals [85]. In turn, *NR2F2* induces the expression of RARβ through RA and RARα [86]. RARβ is considered a tumor suppressor in epithelial cells [87,88]. For example, the expression of RARβ was downregulated in lung tumor tissues, suggesting a potential tumor-suppressive role of RARβ [89,90,91]. Nevertheless, the upregulation of RARβ is also observed in lung cancer tissues [92]. Therefore, further investigation into the roles of RARs and their potential interactions with the NR2F family in lung cancer is warranted.

### 5.4. Retinoic X Receptors (RXRs)

RXRs belong to Class II of the nuclear receptor superfamily and serve as receptors for 9-cis-retinoic acid. RXRs are mainly divided into RXRα (*NR2B1*), RXRβ (*NR2B2*), and RXRγ (*NR2B3*), and RXRγ can further be subdivided into RXRγ1 and RXRγ2 [93]. RXRs can form heterodimers with several nuclear receptor families, including the NR2F family [10,94]. Studies have shown the downregulation of RXRs in lung cancer tissues [95]. Treatment with RXRs agonists, such as bexarotene, inhibits tumor angiogenesis, suppresses the proliferation and migration of lung tumor cells, and promotes tumor cell death through the PPARγ, PTEN, and mTOR pathways [96].

### 5.5. Peroxisome-Proliferator-Activated Receptors (PPARs)

PPARs belong to Class I of the nuclear receptor superfamily and are receptors for fatty acids. PPARs have three subtypes: PPARα, PPARβ, and PPARγ. They form heterodimers to bind onto the peroxisome proliferator response elements (PPREs) on target genes. PPARs are prominently expressed in adipocytes, and the Wnt/β-catenin signaling pathway can increase the expression of *NR2F2* to inhibit PPARγ expression, leading to the suppression of adipogenesis [97]. In addition, a significant decrease of PPARγ expression was reported in lung cancer research [98]. Treatment with PPARγ ligands in adenocarcinoma cell lines inhibits cell proliferation, suggesting that PPARγ ligands hold promise as potential therapeutic agents [99].

### 5.6. Vitamin D Receptors (VDRs)

Vitamin D is synthesized by cells of the immune system and plays a critical role in anti-proliferative activities in cancer cells, such as breast, colon, and stomach tumor cells. VDRs are steroid hormone receptors that induce a cascade of cell signaling to maintain healthy Ca^2+^ levels, which serve to control several biological processes. The NR2F family may compete with VDRs to bind to elements of the VDRs, such as DR3, on their target genes to inhibit the activity of VDRs [10]. The expression levels of VDRs in lung cancer tissues are higher than those in non-cancerous tissues [100]. The expression of VDRs was also associated with improved survival in another lung cancer study [101], suggesting that the dysregulation of VDRs may interact with the NR2F family, leading to malignant transformation in the lungs.

### 5.7. Thyroid Hormone Receptors (TRs)

TRs belong to Class I of the nuclear receptor superfamily and act as receptors for thyroid hormone. TRs consist of two subtypes, TRα and TRβ, which are important regulators of many fundamental physiological processes, including development, growth, and metabolism. The NR2F family inhibits the activities of TRs on their target genes by competing for the TRs’ binding sites [10]. TRα is significantly higher expressed in LUSC than in LUAD, indicating that it may play a dominant role in LUSC [102]. Intriguingly, both types of lung cancer patients exhibit the loss of TRβ expression [103], demonstrating that TRs play diverse roles in different subtypes of lung cancer.

## 6. Discussion

The NR2F family not only plays a role in lung development but also contributes to various lung-related diseases, such as CDH, IPF, and lymphangioleiomyomatosis. Moreover, the NR2F family is essential for the progression of both primary lung cancer and metastatic lung cancer. In primary lung cancer, *NR2F1* and *NR2F2* influence the migration and invasion of tumor cells, while *NR2F6* acts as an immune checkpoint factor to modulate immune processes. In metastatic lung cancer, *NR2F1* mainly inhibits the transition of dormant tumor cells to a proliferative state in the lungs, while *NR2F2* influences tumor cell metastasis to the lungs by affecting the tumor microenvironment, such as angiogenesis or EMT. *NR2F1-AS1* is closely linked to NR2F1-related functions in the progress of lung cancer. Previous studies have provided some preliminary insights into the regulatory mechanisms of the NR2F family in lung cancer and other lung diseases; nonetheless, how the NR2F family participates in the regulation of the tumor microenvironment in lung cancer is still largely unclear. The roles of lung cancer-related genes, such as *KRAS*, *EGFR*, and *ALK*, have been systematically investigated in specific animal models of cancers [104]. However, previous studies on the NR2F family in the lungs have mostly been conducted using lung cancer cell lines or clinical tissue samples. The generation of specific lung cancer animal models for *NR2F1*, *NR2F2*, and *NR2F6* will not only enhance the understanding of the molecular mechanisms of lung cancer but also improve the diagnosis and therapy for lung cancer associated with NR2F family dysregulation.

Previous studies have demonstrated that nuclear receptors are excellent targets for cancer therapy. Currently, drugs against nuclear receptors, such as ER and RXR, have been developed and used to treat various cancers, including breast cancer, with convincing efficacy [105]. The NR2F nuclear receptor subfamily, which interacts with various nuclear receptors, including RARs and RXRs, is a potential novel therapeutic target in cancer, especially in lung cancer.

Single-cell and spatial omics technologies are rapidly advancing and have been widely applied to research on development and disease, which enables precise identification of cellular heterogeneity and cell–cell communications. Spatial omics technology, which can simultaneously provide spatial location and omics information of tissues, makes it possible to uncover the interactions among cells in the tumor microenvironment [106]. Recently, spatial omics methods have been used to compare the difference between primary and metastatic tumor tissues in the brain metastasis of NSCLC. Changes in the immune-suppressive and fibrotic microenvironment were identified, and those changes aid the metastatic tumor cells in creating a suitable niche for rapid proliferation and progression in the brain [107].

In summary, the expression and functions of the NR2F family are closely associated with lung development and lung-related diseases. By establishing well-designed animal models targeting the NR2F family members in the lungs and combining the latest technologies, like spatial omics, a better understanding of the molecular and cellular mechanisms of the NR2F family in the development and diseases of the lung may be achieved, which will benefit the findings of novel diagnostic and therapeutic approaches for NR2F-related lung diseases, including lung cancer.

## Figures and Tables

**Table 1:** Primary lung diseases related to the NR2F family.

Disease Type		Genes	Functions	Models/Cell Lines/Tissues	Related Genes	Related Pathways	Reference
Non-cancerous	CDH	*Nr2f2*↑	May rescue lung hypoplasia and enhance lung growth	Nitrofen rat model of CDH	*Fog2* and *Gata4*	-	[57]
		*Nr2f2*↓	Formation of CDH	Nkx3-2^Cre/+^; Nr2f2^flox/flox^ mouse model	*Fog2*	-	[21]
		*NR2F2*↓	Formation of CDH	15q deletion patients specimens	*CHD2*, *RGMA* and *SIAT8B*	-	[43]
	IPF	*Nr2f2*↑	Decreases fibrosis	Bleomycin-treated mice model	*Fn1* and *Col1a1*	-	[44]
	LAM	*NR2F2*↑	Drives LAM pathogenesis	S-LAM patients specimens	*MCTP2* and *SPATA8*	-	[46]
Cancerous	NSCLC	*NR2F1-AS1*↓	Decrease NSCLC cell proliferation, migration, and invasion and promoted tumor cell apoptosis	NSCLC patients specimens; BEAS-2B, H522, H460, H1299, A549 and SK-MES-1 cell lines; nude mice	-	NR2F1-AS1/miR-493-5p/ITGB1 pathway	[58]
	NSCLC	*NR2F1-AS1*↑	Tumorigenic, promotes glycolysis and glutamine metabolism	NSCLC patients’ specimens; 16HBE, A549 and H522 cells	-	miR-363–3p/SOX4 axis	[59]
	LUAD	*NR2F6*↑	Promote proliferation, migration, invasion and enhances cell apoptosis	Lung adenocarcinoma patients specimens; A549, HCC827, HBE cells	*miR-142-3p*	-	[54]
	Lung Carcinoma	*NR2F2*↑	Promote cell invasion	A549, HeLa, NCI-H460, H661, H520, H441, MDAMB231 and H460SMcells	*FAK(PTK2)*, *MMP2*, *uPA* and *uPAR*	-	[52]
	LUAD	*NR2F1*↑	Promote growth, migration, invasion, and tumorigenicity of lung adenocarcinoma cells	393P, 344SQ, 412P, 307P, 344LN, 344P, 393LN, 531LN1, 531LN2, 531LN3, 531P1, 531P2, 713P, A549 and HCC827 cells	*ZEB1*	ZEB1/NR2F1/NR2F1-AS1 axis	[51]
	LUAD	*NR2F2*↑	Induces platinum chemotherapeutic resistance in lung cancer brain metastasis	PC9, PC9-BrM1 and PC9-BrM3 cells; Nude mice	*GSTM1* and *GPX4*	Wnt signaling pathway	[53]

↑, upregulation; ↓, downregulation.

**Table 2:** Metastatic lung cancer related to the NR2F family.

Primary Cancer Types	Genes	Inhibition/Promotion Metastasis	Models/Cell Lines/Tissues	Related Genes	Related Pathways	Reference
Breast cancer	*NR2F1-AS1*↑	Inhibition	BALB/c nude mice and NOD/SCID mice; CA1h-P1, CA1h-P2 and 4175-LM2 cells	*PTBP* and *miR-205*	NR2F1/ΔNp63 axis	[66]
Pancreatic cancer	*NR2F1-AS1*↑	Promotion	PC and matched paracancerous tissue samples; BxPC-3, Capan-2, CFPAC-1, SW1990, MIA PaCa-2, PANC-1 and HPDE cells; nude mouse	*NR2F1*	HIF pathway, AKT/mTOR pathway	[69]
SACC	*NR2F1*↑	Inhibition	SACC patients specimens; SACC-83 and SACC-LM cells; nude mice	-	CXCL12/ CXCR4 pathway	[67]
HNSCC	*NR2F1*↑	Inhibition	T-HEp3 cells and D-HEp3 cells; chicken chorioallantoic membrane (CAM) model; NU/J female mice model	-	TGF-β/SMAD4 signaling pathway	[65]
Gastric cancer	*Nr2f2*↓	Inhibition	Gastric cancer patients specimens; SGC-7901, BGC-823, MGC-803, MKN-45, MKN-28 and AGS cell lines; nude mice	*Fbxo21* and *Zeb1*	Nr2f2/Snail pathway	[68]
Breast carcinoma	*Nr2f2*↓	Inhibition	ROSA26^CRE-ERT2/+^; Nr2f2^flox/flox^mouse model and PyMT^+/−^/ROSA26^CRE-ERT2/+^; Nr2f2^flox/flox^ mouse model; B16F10 and LLC cells	*Ang-1*	VEGF signaling pathway	[24]

↑, upregulation; ↓, downregulation.

## Data Availability

Not applicable.
